# Antioxidants Impair Anti-Tumoral Effects of Vorinostat, but Not Anti-Neoplastic Effects of Vorinostat and Caspase-8 Downregulation

**DOI:** 10.1371/journal.pone.0092764

**Published:** 2014-03-20

**Authors:** Laura Bergadà, Andree Yeramian, Annabel Sorolla, Xavier Matias-Guiu, Xavier Dolcet

**Affiliations:** Oncologic Pathology Group, Department de Ciències Mèdiques Bàsiques, Universitat de Lleida, Hospital Universitari Arnau de Vilanova, Institut de Recerca Biomèdica de Lleida, IRBLleida, Lleida, Spain; Universitat de Lleida – IRBLLEIDA, Spain

## Abstract

We have recently demonstrated that histone deacetylase inhibitor, Vorinostat, applied as a single therapy or in combination with caspase-8 downregulation exhibits high anti-tumoral activity on endometrial carcinoma cell lines. In the present study, we have assessed the signalling processes underlying anti-tumoral effects of Vorinostat. Increasing evidence suggests that reactive oxygen species are responsible for histone deacetylase inhibitor-induced cell killing. We have found that Vorinostat induces formation of reactive oxygen species and DNA damage. To investigate the role of oxidative stress as anti-neoplastic mechanism, we have evaluated the effects of different antioxidants (Bha, Nac and Tiron) on endometrial carcinoma cell line Ishikawa treated with Vorinostat. We show that Bha, Nac and Tiron markedly inhibited the cytotoxic effects of Vorinostat, increasing cell viability in vitro. We found that all three antioxidants did not inhibited accumulation of acetyl Histone H4, so that antioxidants did not inhibit Vorinostat activity. Finally, we have evaluated the effects of antioxidants on anti-tumoral activity of Vorinostat as monotherapy or in combination with caspase-8 downregulation *in vivo*. Interestingly, antioxidants blocked the reduction of tumour growth caused by Vorinostat, but they were unable to inhibit anti-tumoral activity of Vorinostat plus caspase-8 inhibition.

## Introduction

Histone deacetylase (HDAC) and histone acetyltransferase (HAT) are a class of enzymes which regulate the acetylation status of histones and non-histone proteins, the most frequently post-translational epigenetic modification in eukaryotic cells [1], [2]. Histone tails are normally positively charged due to amine groups. These positive charges help them to interact with and bind to the negatively charged phosphate groups on the DNA backbone. HATs induce protein acetylation that neutralizes the positive charges on the histone by changing amines into amides decreasing the ability of the histones to bind to DNA. This allows chromatin expansion and gene transcription. HDACs induce protein hypoacetylation increasing positive charge of histone tails causing compaction of the DNA/histone complex and preventing expression of genes required for cell cycle arrest and apoptosis [3]–[7]. Aberrant protein acetylation, mostly on histones, has been related to cancer while anomalous expression of HDACs has been found in large types of cancer [8], [9]. One of the most promising groups of anticancer agents is the inhibitors of histone deacetylases (HDACis), that lead to an accumulation of acetylated proteins both in tumor and normal cells [10]–[12]. Therefore, HDACi have been found to suppress migration and invasion, cause growth arrest, differentiation, and apoptosis in a broad types of cancers such as thyroid [13], [14], breast [15], [16], colon [17], [18], endometrial [19], [20], ovary [20],lung [21], [22] and others by altering the transcription of a small number of genes [9]–[12],[23]. A recent study of our laboratory has demonstrated that HDACi such as Vorinostat can also be effective for inducing apoptotic cell death in endometrial cancer (EC) cells [19]. In this previous work, we have demonstrated that Vorinostat displays anti-neoplastic activity on EC cells alone or in combination with inhibition of caspase-8.

Several mechanisms underlying anti-neoplastic activity of Vorinostat have been proposed, including induction of apoptotic pathways, cell growth arrest, autophagy, mitotic catastrophe, or senescence [24]–[26]. Some of these processes such as apoptosis or cell growth are affected by multiple intracellular events, including the production of reactive oxygen species (ROS) [24]–[26]. Increasing lines of evidence demonstrate that, in addition to their effects on histone acetylation and epigenetic regulation, production of ROS may participate in anti-tumoral activity of HDACis. Recent studies suggest that HDACis might cause cell death by the induction of ROS such as superoxide, hydroxyl radical and hydrogen peroxide as determined by DCF dye oxidation assay in a large variety of cancers [27]–[30]. Along with ROS generation, DNA damage has usually been observed during the process of cell death [31], [32]. These two phenomena, the increase of ROS level and DNA damage, can be found either independent or one being caused by the other one [33].

In the present study, we have investigated the mechanisms by which Vorinostat exerts its anti-tumoral activity on EC. We have concentrated the study on the effects of Vorinostat on ROS production and we have analysed whether ROS production high oxidative stress is involved in cytotoxic effects of Vorinostat alone or in combination with caspase-8 inhibition.

## Materials and Methods

### Cell Lines, Culture Conditions and Transfection

The Ishikawa 3-H-12 cell line (IK) was obtained from the American Type Culture Collection (Manassas, VA). EC cells were grown in Dulbecco's modified Eagle's medium (Sigma, St. Louis, MO) supplemented with 10% fetal bovine serum (Invitrogen, Inc., Carlsbad, CA), 1 mmol/L HEPES (Sigma), 1 mmol/L sodium pyruvate (Sigma), 2 mmol/L L-glutamine (Sigma), and 1% of penicillin/streptomycin (Sigma) at 37°C with saturating humidity and 5% CO_2_.

### Chemical reagents

Vorinostat (suberoylanilide hydroxamic acid) was obtained from Merck. A 50 mM stock was prepared in dimethylsulphoxide (DMSO) and stored at −80°C. 2,5- diphenyl tetrazolium bromide assay (MTT), DMSO, bis-benzimide fluorescent dye (Hoechst 33258), Butylated hydroxyanisole (BHA; *tert*-butyl-4-hydroxyanisole), N-Acetyl Cysteine (Nac), Sodium 4,5-dihydroxybenzene-1,3-disulfonate (Tiron), Vitamin C, Rodamine 123 (Rh123), fluorescent probe CM-H2DCFDA, DHE (D7008-10 mg) and monoclonal antibody to Tubulin were from Sigma (St Louis, MO, USA).

### Cell Viability Assays and Assessment of Apoptosis

The general mitochondrial activity of EC cell lines was determined by assaying reduction of MTT (3-(4, 5-dimethylthiazol-2-yl)-2, 5 diphenyltetrazolium bromide) to formazan. EC cells were plated on M96-well plates at 15×10^3^ cells per well. After the indicated treatments, cells were incubated for 30 minutes with 0.5 mg/ml of MTT reagent and lysed with dimethylsulphoxide (DMSO) to dissolve the blue formazan crystals produced by the mitochondrial succinate dehydrogenase of the living cells. Cell viability proportionate to optical density was measured using a colorimetric assay of mitochondria activity. Drug resistance was represented as the percentage of live cells surviving after drug treatment relative to control cells. Absorbance were measured using a spectrophotometer (Bio-Rad, Richmond, CA, U.S.A.) at a dual wavelength of 595 nm and 620 nm.

Apoptotic cells were identified by nuclear staining with bis-benzimide fluorescent dye (Hoechst 33258), after the indicated treatments, to a final concentration of 0.5 mg/ml to each M24 well. Cells were counted under epifluorescence microscope (Leica Microsystems, Wetzlar, Germany).

### Clonogenic assay

EC cells were seeded onto 6-well plates at a density of 1×10^3^ cells per dish. On the next day, cells were treated for 24 hours, after that period of time mediums were changed and they were incubated for 12–14 days. The colonies were stained with MTT (1/10) for 30 minutes and fixed in methanol for 5 minutes and maintained in 2 mL PBS. Colonies consisting of >50 cells were scored using the Quantity One program (Colony Counting Quick Guide) and two replicate dishes were counted for each treatment.

### Western Blot Analysis

The EC cell lines was washed with cold phosphate-buffered saline (PBS) and lysed with lysis buffer (2% sodium dodecyl sulfate, 125 mmol/L Tris-HCl, pH 6.8). Protein concentrations were determined with a protein assay kit (Bio-Rad, Hercules, CA). Equal amounts of proteins were subjected to sodium dodecyl sulfate-polyacrylamide gel electrophoresis and transferred to polyvinylidene difluoride membranes (Millipore). Nonspecific binding was blocked by incubation with TBST (20 mmol/L Tris-HCl, pH 7.4, 150 mmol/L NaCl, 0.1% Tween-20) plus 5% of nonfat milk. Membranes were incubated with the primary antibodies overnight at 4°C. Signal was detected with ECL Advance (Amersham-Pharmacia, Buckinghamshire, UK). The antibodies used were: active caspase-3 and caspase-9 were obtained from Cell-Signal (Beverly, MA), active caspase-8 (monoclonal, Calbiochem), anti-phospho-Histone H2A.X (Ser139) (monoclonal, Millipore), phosphor-38 (Thr180/Tyr182) (polyclonal, Calbiochem), Anti-p38 MAPKinse (pThr 180, pTyr 182) (polyclonal, Calbiochem), p21 (monoclonal, Cell-Signal), p27 (monoclonal, Cell-Signal), α-tubulin (monoclonal, Sigma) and anti-Acetyl-Histone H4 (Lys12) (polyclonal, Cell-Signal) were used as a Vorinostat control.

Membranes were then incubated with peroxidase-coupled anti-mouse or anti-rabbit secondary antibodies (Amersham-Pharmacia Uppsala, Sweden) for 1 h, followed by chemiluminescent detection with ECL Advance (Amersham Pharmacia, Buckinghamshire, UK).

The acetyl histone H4 band intensities were calculated and normalized to Tubulin intensities using Image Lab 4.0.1. Lane and Bands Quantity Analysis Tools.

### Measurements of intracellular reactive oxygen species (ROS)

Dichorofluorescein diacetate (DCFH-DA) was used to assess levels of net intracellular generation of ROS and dihydroethidium (DHE), was used to determine levels of superoxide anion (.O_2_^−^). DCFH-DA is a peroxidesensitive fluorescent probe that is nonpolar and diffuses into the cell. Intracellular esterases cleave the diacetate ester group and entrap the polar, nonfluorescent DCFH within the cell. ROS can oxidize this substance to the fluorescent compound DCF. As a positive control, IK cells were incubated with hydrogen peroxide (H_2_O_2_) (1 mM) for 30 min prior to the lost their ability to entrap the DCFH-DA dye and convert it to the membrane impermeable form DCF (a function of their intracellular esterase activity). Cells were treated with Vorinostat for the indicated times, washed twice in PBS, tripsinized and 6×10^6^ ml cells were incubated in PBS containing 20 μM DCFH-DA or 10 μM DHE for 30 min prior to ROS measurement. The fluorescence of the cell population is proportional to the levels of intracellular ROS generated, and was measured with a FACScan (Becton Dickinson, Mountain View, CA, USA) at 588 nm emission.

### Immunofluorescence and quantification of γH2AX

After indicated doses of treatment, IK cells were maintained at 37°C and 5%CO2 until fixation at 24 hours post-treatment. Cells were washed twice with phosphate-buffered saline (PBS), fixed for 20 min with 4% paraformaldehyde, permeabilized with methanol during 5 min, washed and maintained with PBS. After blocking (5% HS +5% FBS +0,2% Glicina +0,1% Tritó in PBS) during 1 h, cells were incubated overnight at 4°C with a monoclonal anti-*γ*H2AX antibody (monoclonal, Millipore), anti-Acetyl-Histone H4 (Lys12) (polyclonal, Cell-Signal) 1∶500 in PBS, and washed and incubated with FITClabeled secondary antibody (Sigma) 1∶500 in PBS in the dark for 1 h at room temperature. Cells were then washed, counterstained, and stained with bis-benzimide fluorescent dye (Hoechst 33258), to a final concentration of 0.5 mg/ml, in the dark for 35 minutes. Cells were counted under epifluorescence microscope (Leica Microsystems, Wetzlar, Germany). Aleatory sampling methods were used to select the images and all the cells in each selected image were screened. The *γ*H2AX foci per nucleus were counted by eye. Cells were judged as “positive” for *γ*H2AX foci if they displayed 10 or more discrete dots of brightness. For quantitation of foci, a minimum of 300 cells were analysed for each time point.

### Xenografting and Vorinostat/Nac administration

Immunodeficient female SCID hr/hr mice (age, 12 weeks; weight 20–25 g) were maintained in Specific Pathogen Free (SPF) conditions. Animals were subcutaneously injected with HEC-1A cells (1,5×10^6^) suspended in 100 μl PBS+Matrigel (1∶1). Tumors were allowed to growth for 38 days. Xenografted mice were treated with an intraperitoneal injection of Vorinostat at 50 mg/kg/day and with or without Nac 250 mg/kg/day for 5 days/week during two consecutive weeks. Tumors were measured weekly with calipers. Tumor size was calculated using the formula: Tumor weight (TW, mg) = (D*d2)/2 = mm3.

## Results

### Vorinostat induces ROS production and DNA damage on endometrial cancer cells

**Figure 1:**
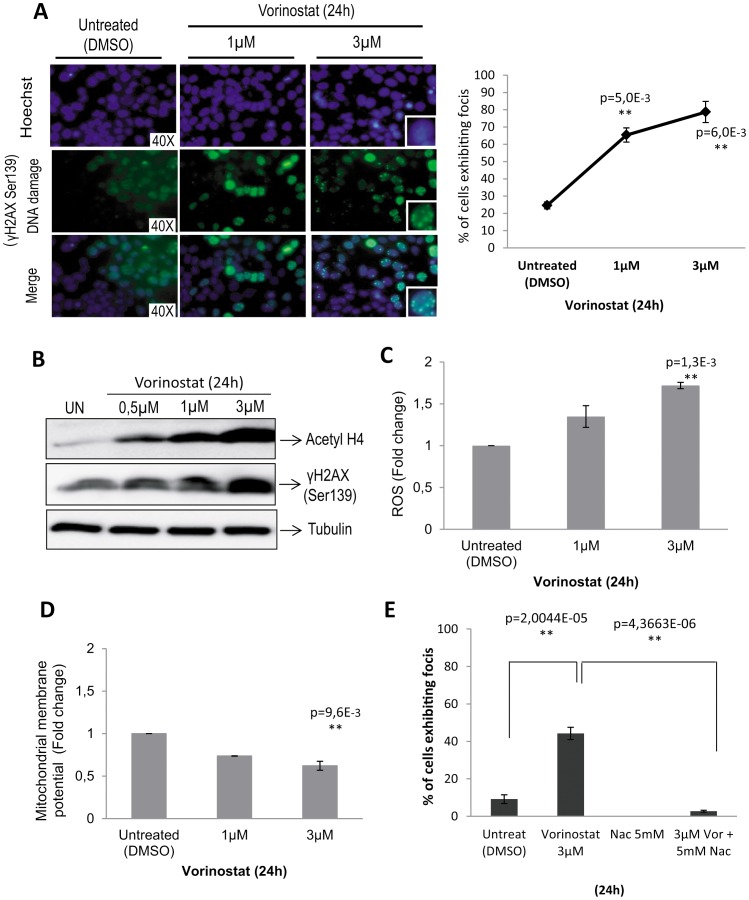
Vorinostat induces DNA damage and production of ROS in IK cell line. A) Representative images (left) and quantification (right) of γH2AX immunofluorescence of IK cells showing foci formation after Vorinostat treatment. **p<0.001 compared with the control group. B) Ik cells were treated with the indicated doses of Vorinostat and cell lysates were subjected to western blot using antibodies anti-acetylated H4, anti-H2AX (Ser-139) and antitubulin. C) Quantification of ROS production determined by staining with DCFH-DA and quantified by flow cytometer (Left graph). D) Loss of mitochondrial membrane potential (MMP) was determined by staining with Rhodamine 123 and quantified by flow cytometer (Right graph). An absence of Rho123 from cells indicated the loss of MMP (ΔΨ_m_). **p<0.001 compared with the control group. E) Quantification of ROS production on cells treated with Vorinostat or Vorinostat plus the anti-oxidant NaC. **p<0.001 compared with the control group.

We have recently demonstrated that Vorinostat is an effective anti-neoplastic agent for EC cell lines [19]. Here, we have investigated the molecular mechanisms of anti-neoplastic effects of Vorinostat. Since Vorinostat is an HDAC inhibitor, we first investigated the ability of Vorinostat to cause DNA damage on the EC cell line IK. For this purpose, we measured Vorinostat-induced γH2AX foci formation by immunofluorescence microscopy (Fig 1A). Vorinostat caused a significant increase of nuclear γH2AX foci 24 hours after treatment. In control IK cells, the number of foci per nucleus averaged 24.5% and increased to 65.4% and 78.7% after treatment with 1 μM or 3 μM, respectively. Increased phosphorylation of γH2AX was further confirmed by western blot analysis of IK cells treated with Vorinostat for 24 hours (Fig 1B). These findings suggest that Vorinostat caused double strand DNA breaks on IK cells.

Previous studies have suggested that the lethal effect of HDACis could be related to oxidative stress as measured by the increase of ROS levels [27]–[30]. Generation of ROS in IK cells was monitored during the treatment with Vorinostat at different concentrations (1 and 3 μM) (Fig 1C). ROS concentration was substantially increased in Vorinostat treated cells. Such increase correlated with a loss of membrane potential (MMP) (as measured by Rho123 staining) (Fig 1D). These results indicate that Vorinostat treatment results in increased of oxidative stress in IK cells.

Finally, to demonstrate specific production of ROS after Vorinostat treatment, IK cells were exposed to 5 mM Nac plus 3 μM Vorinostat for 24 h. These IK cells were harvested for the determination of ROS production. To evaluate the general profiles of ROS production in these cells, CM-H_2_DCFDA was used in the assays for the detection of intracellular superoxide, hydrogen peroxide, and other ROS. The co-treatment of IK cells with Nac alleviated Vorinostat-induced ROS production, compared with single-agent treatment (Fig 1E). IK cells treated for 30 min with H_2_O_2_ were assumed as positive control.

### Antioxidants inhibit the cytotoxic effects and the apoptotic features induced by Vorinostat treatment

**Figure 2:**
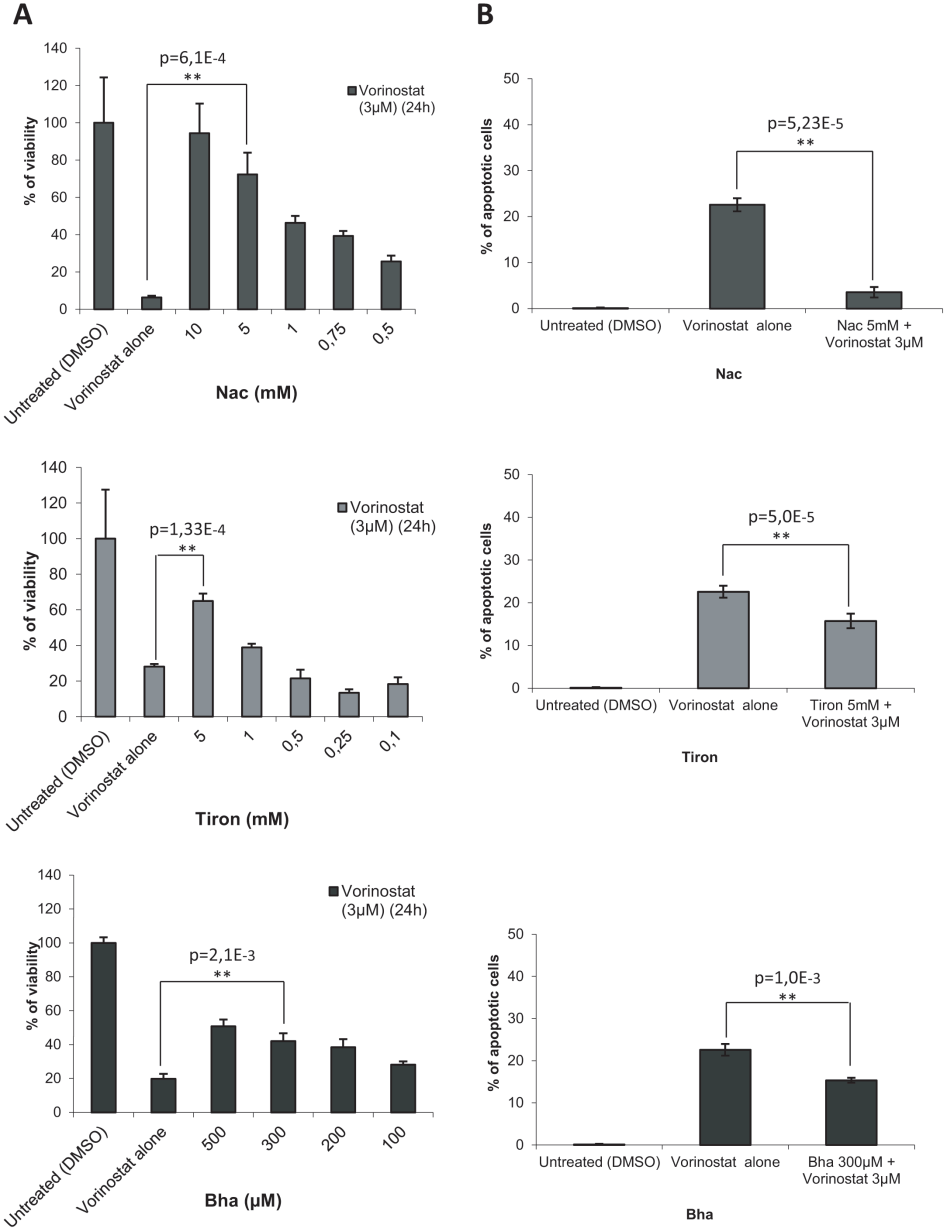
Effects of Nac, Tiron and Bha on reduction of cell viability caused by Vorinostat. A) IK cells were treated with 3 μM Vorinostat, alone or in combination with increasing doses of Nac, Tiron and Bha. Cell viability was assessed by MTT assay 24 h after treatment. Results are expressed as percent of viability. B) Quantification of Hoechst-stained apoptotic nuclei of IK cells was realized 24 h after being treated with Vorinostat, Vorinostat plus antioxidants or left untreated. Results are expressed as percent of the control values.

Once demonstrated that ROS production is involved in Vorinostat-induced DNA damage on EC cells, we sought to investigate their role in anti-neoplastic activity of Vorinostat. For this purpose, we first tested the effects of three different antioxidants (Nac, Bha and Tiron) on the decrease in cell viability caused by Vorinostat treatment. IK cell line was treated with Vorinostat 3 μM plus increasing doses of the three antioxidants and cell viability was assessed by MTT assay 24 h after treatment. The results showed that all three antioxidants caused a dose-dependent inhibition of Vorinostat cytotoxicity (Fig 2A). We also addressed the effects of Nac, Bha and Tiron on apoptotic nuclear morphology caused by Vorinostat. For this purpose, IK cells were treated with 3 μM Vorinostat, alone or in the presence of 5 mM Nac, 300 μM Bha and 5 mM Tiron for 24 hours. Cells were stained with Hoechst and the number of nuclei displaying apoptotic morphology was evaluated. Addition of Nac dramatically reduced the number of nuclei displaying apoptotic morphology in the cultures treated with Vorinostat (Fig 2B). Addition of Bha and Tiron also reduced the number of apoptotic cells, although these two antioxidant were less effective than Nac (Fig2B). Because Nac displayed highest efficiency in reducing pro-apoptotic effects of Vorinostat, we chose it for subsequent experiments.

Collectively, these findings indicate that ROS production caused by Vorinostat plays a significant functional role in the enhanced lethality induced by Vorinostat treatment.

### Co-administration of Nac and Vorinostat reduces DNA damage and ROS production

**Figure 3:**
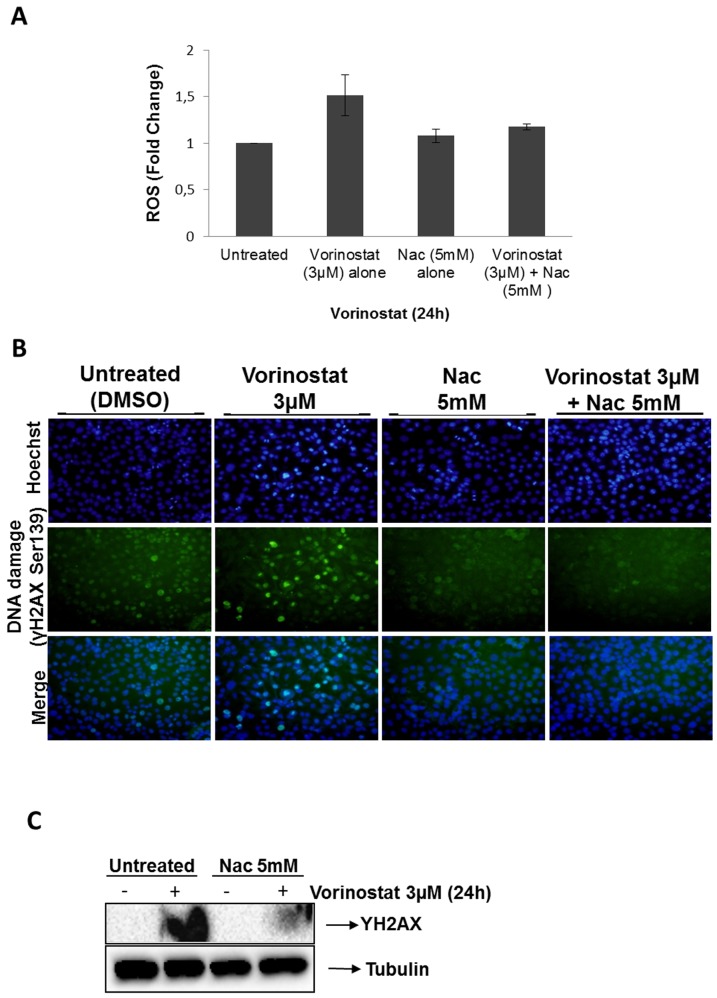
Nac treatment reduces DNA damage and ROS production in IK endometrial cell line. A) Representative γH2AXimmunofluorescence corresponding to IK cells after the combination of Vorinostat and Nac treatment at indicated time. Cells were stained with Hoechst to evidence nuclei. B). Quantifification of the number of cells exhibiting γH2AX positive focis. **p<0.001 compared with the control group C) IK cells were treated with Vorinostat plus Nac, at indicate doses, or left untreated for 24 h. Cell lysates were subjected to western blot with, anti-H2AX (Ser-139) and anti-Tubulin.

To evaluate the effects of Nac in Vorinostat-induce DNA damage, we determined the number of γH2AX foci in IK cells upon co-treatment with Nac plus Vorinostat. The number of cells displaying focis in their nucleus was dramatically decreased in cell cultures co-treated with Vorinostat plus Nac (Fig A, 3B). As shown in Figure 3B, the induction of γH2AX in IK cell line was observed after 24 h, indicating an evident potentiation by Vorinostat-induced DNA damage. In contrast, cotreatment of IK cells with Nac plus Vorinostat results in a decrease of H2AX phosphorylation (Fig 3C). These findings indicate that treatment of IK cells with Vorinostat potently induces DNA damage associated with caspase-dependent activation of the apoptotic program. Collectively, these findings suggest that oxidative injury plays a significant functional role in lethality induced the Vorinostat in IK cancer cells.

### Antioxidants do not impair Vorinostat-induced Acetyl-H4 accumulation

**Figure 4:**
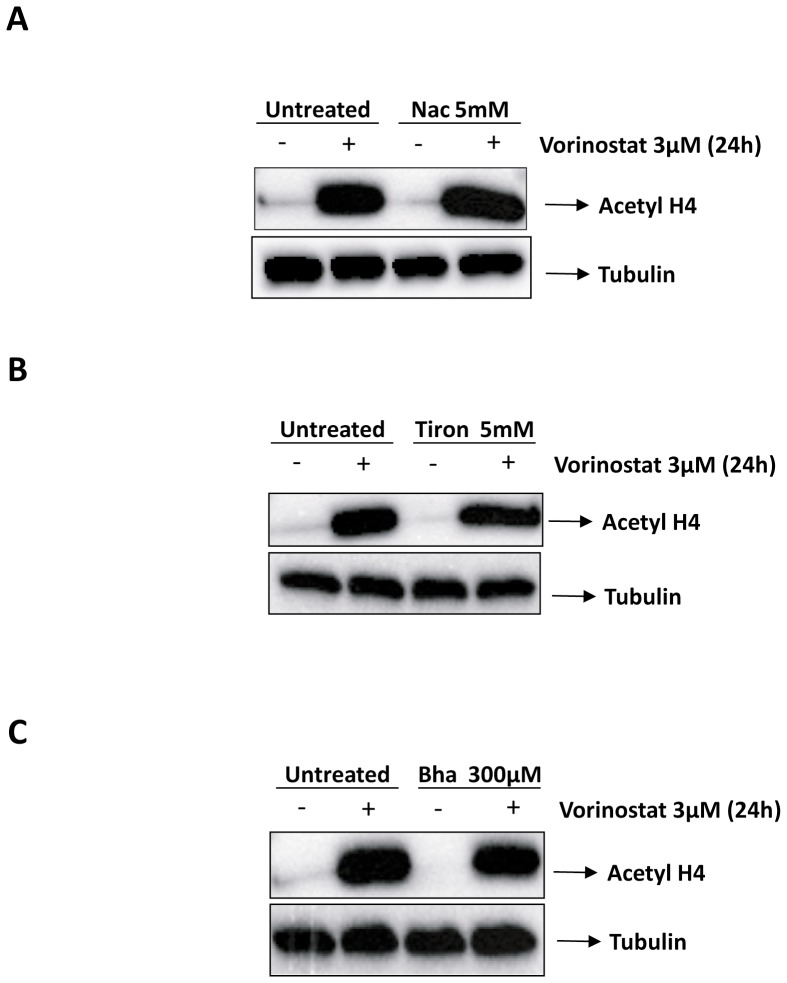
Effect of co-treatment with Vorinostat and antioxidants on histone acetylation. Representative western blot showing acetylated histone H4 and tubulin western blot analysis of IK cells treated or left untreated with 3 μM Vorinostat, IK cells treated with indicated doses of Nac, Tiron and Bha antioxidants and finally, IK cells treated with Vorinostat together with antioxidants at indicated doses for 24 hours. Mean and standard deviations of the ratios of intensities of Ac-histone H4 band-Tubulin bands from the corresponding lanes.

We have previously reported that antioxidants can block the activity of antineoplastic drugs by interfering with their mechanism of action [34]. Because antioxidants markedly reduced DNA damage and ROS production, we hypothesized that antioxidants could be affecting the ability of Vorinostat to modify histone acetylation. To rule out this possibility, we monitored histone H4 acetylation after co-treatment of IK cells with Vorinostat plus antioxidants. To ensure that Vorinostat treatment was effective when we administrated antioxidants, we first examined the accumulation of Acetyl H4 by Western Blot. Accumulation of Acetyl H4 results from the histone hyperacetylation caused by the activity of HDACi. IK cells treated with Nac, Bha or Tiron plus Vorinostat, showed similar amounts of Acetylated H4 products to those observed in cells treated with Vorinostat alone (Figure 4A, B and C, see lanes 2 and 4). These results demonstrate that antioxidants do not impair the molecular mechanism of Vorinostat's action.

### Co-treatment with Nac and Vorinostat increased the colony capacity and reduced the cleavage of caspase-3

**Figure 5:**
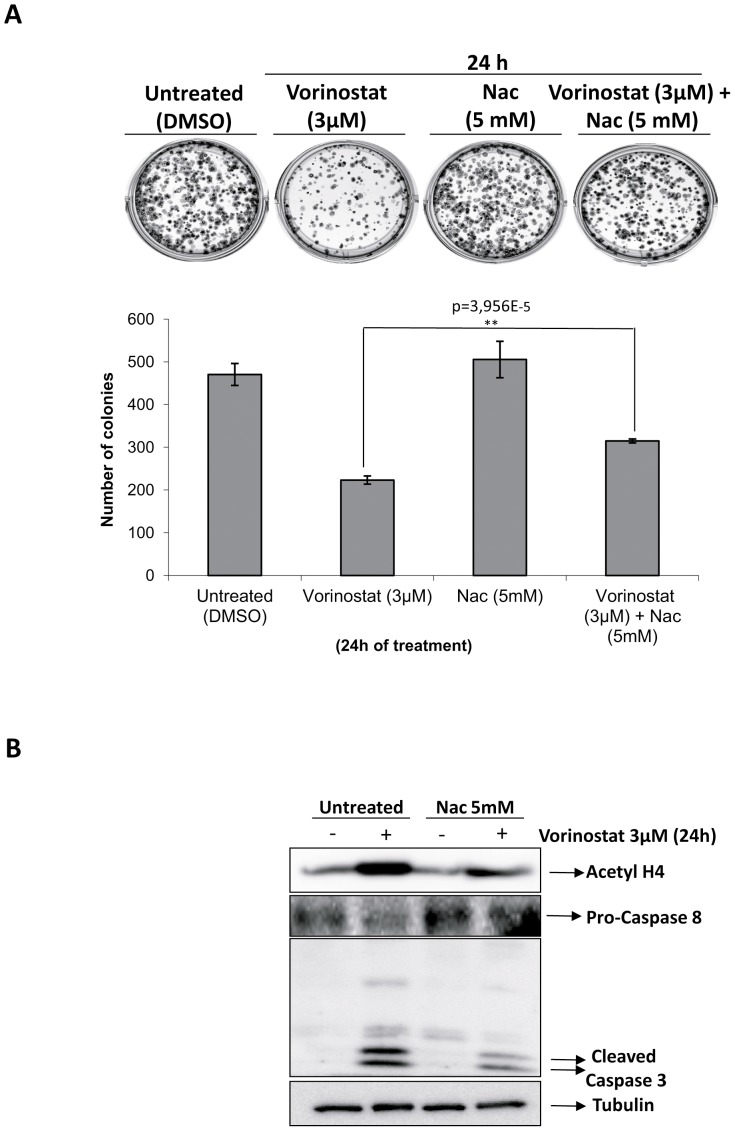
Nac treatment increases colony formation and reduces caspase-3 cleavage in IK endometrial cancer cells treated with Vorinostat. A) IK cells were seeded as a single-cell suspension with a specified number of cells. After allowing cells time to attach (6 h), Vorinostat plus Nac or the vehicle control was added and treated for indicated times. Ten to fourteen days after seeding, survival curves (from counting the number of colonies) were generated after normalizing. *p<0.05, **p<0.001 compared with the control group. B) IK cells were treated with Vorinostat and Nac, at indicate doses, or left untreated (UN) for 24 h. Cell lysates were subjected to western blot with Acetylate H4, anti-caspase-3, 8 and anti-Tubulin.

Once demonstrated that antioxidants reduced ROS production and DNA damage without interfering with acetylation of histones, we investigated the effects of Nac on con clonogenic capability and apoptosis of IK cells treated with Vorinostat. Combination of Vorinostat plus Nac treatment resulted in a significant increase of colony cells exhibiting surviving fractions over cultures treated with Vorinostat alone (p-value = 3,956* 10^−5^) (Fig 5A). Western blot analysis (Fig 5B) revealed that co-administration of Vorinostat and Nac resulted in a decrease in caspase-3 processing. These results suggest that Nac co-treatment reduces Vorinostat-induced cell death and clonogenic ability, suggesting that ROS production was plays a critical role in mediating anti-tumoral effects of Vorinostat.

### Nac treatment does not inhibit antineoplastic effects of Vorinostat plus caspase-8 inhibition

**Figure 6:**
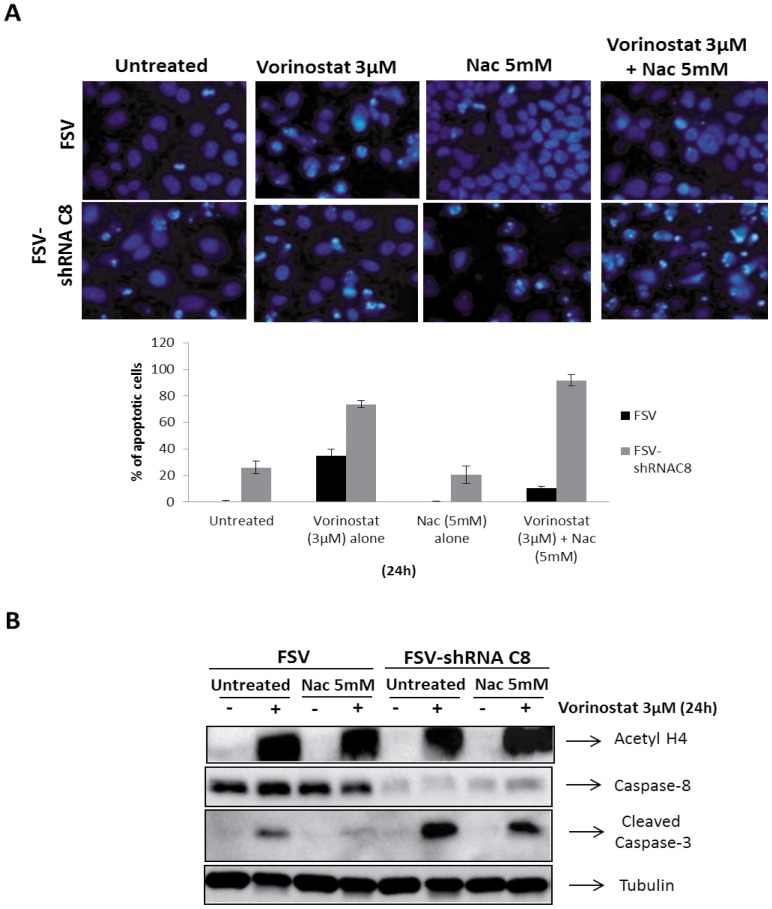
Nac treatment do not reduce the effects of blocking caspase-8 and Vorinostat treatment in IK cancer cell line. A) IK cells were infected with lentiviruses carrying Caspase-8 shRNA (FSV-Casp-8 shRNA) or vehicle control (FSV) and treated with 3 μM Vorinostat plus 5 mM Nac at indicated times. The graph represents a quantification of nuclei displaying nuclear apoptotic morphology. B) Western blot analysis of whole lysates from IK cells infected with lentiviruses carrying Caspase-8 shRNA (FSV-Casp-8 shRNA) or vehicle control (FSV). The membranes were reproved with Acetyl H4, Caspase-8, Caspase-3 and Tubulin to ensure equal protein loading.

We have recently demonstrated that combination of Vorinostat and caspase-8 exhibits high anti-neoplastic activity on EC cells *in vivo* and *in vitro*. Therefore, we wondered whether antioxidants could also block the anti-tumoral effects of caspase-8 inhibition plus Vorinostat combination. For this purpose, IK cells were infected with lentiviruses carrying caspase-8 shRNA and were treated with 3 μM Vorinostat alone or in combination with 5 mM Nac for 24 h. After that period of time we counted the number of cells displaying apoptotic morphology. Interestingly, Nac treatment did not reduce the number of apoptotic cells when caspase-8 was downregulated (Fig 6A). Accordingly, Western Blot analysis revealed that caspase-3 processing was not reduced after Nac treatment when caspase-8 was inhibited. These data indicates that, anti-tumoral action of caspase-8 inhibition plus Vorinostat treatment is insensitive to antioxidants.

### Nac impairs anti-tumoral activity of Vorinostat, but not anti-tumoral effects of Vorinostat plus caspase-8 inhibition

**Figure 7:**
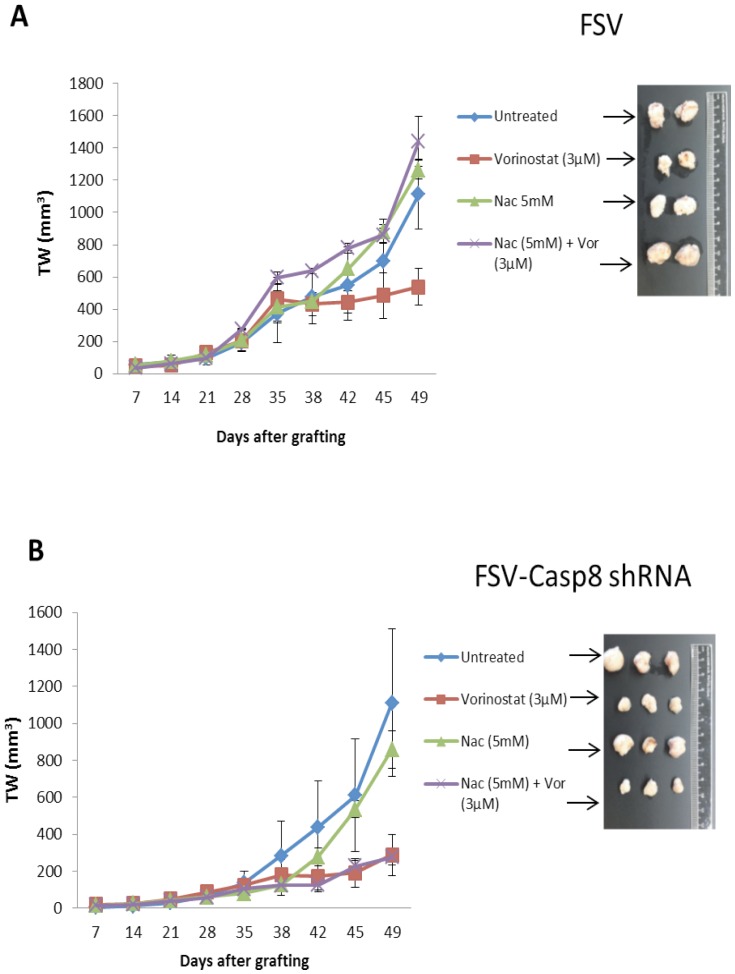
Nac treatment impairs Vorinostat but not Vorinostat plus caspase-8 inhibition effects on endometrial cancer. Tumour weight (TW) measuraments and representative images corresponding to tumours developed by IK cells infected with lentiviruses carrying control vector (FSV) or caspase-8 shRNA (FSV-Casp8 shRNA) and subcutaneously grafted in SCID mice. At day 42, mice were divided into four groups, and were untreated (with vehicle) or injected intraperitoneally with Nac alone (250 mg/kg/day), Vorinostat alone (50 mg/kg/day) or Vorinostat plus Nac. Graphs (A) In the presence of caspase-88, Nac blocks anti-tumor effect of Vorinostat (B). In the absence of caspase-8, Nac does not block anti-tumor effect of Vorinostat.

Finally, we assessed the effects of antioxidants on therapeutic potential of Vorinostat or Vorinostat plus caspase-8 inhibition *in vivo*. For this purpose, IK cells were infected with lentiviruses containing control shRNA (FSV) or caspase-8 shRNA (FSV-Caspase-8) and were xenotransplanted; tumors were allowed to growth for 42 days. At this point, mice were divided in four groups: first group was left untreated (injected with vehicle), a second group was injected with Nac alone, the third group was injected with Vorinostat alone and the last group was injected with Nac and one hour after with Vorinostat. This treatment was applied daily during two weeks (see material and methods for protocol of administration). Tumor size and animal weight was measured weekly from the first day of cell injection to the end of the protocol. As we show in Figure 7A, administration of Vorinostat plus Nac led to development of tumours displaying similar size to that observed in control (without treatment) mice. These results indicate that, as we observed *in vitro*, antioxidants also block anti-tumoral effects of Vorinostat *in vivo*. In contrast, the same experiment performed with IK cells with downregulated caspase-8 had a different outcome. In this case, tumors from Vorinostat or Vorinostat plus Nac treated mice had similar reduction over non treated mice (Fig 7A), indicating that antioxidants are unable to block the effects of Vorinostat plus caspase-8 combination of EC cells (Fig 7B).

## Discussion

The main goal of this study was to address the molecular mechanisms underlying anti-tumoral activity of the HDACi, Vorinostat. We have recently demonstrated that Vorinostat has a marked anti-tumoral activity on endometrial cancer cells, both*in vitro* and *in vivo* [19]. In this previous work, we demonstrated that Vorinostat induced cell growth arrest, loss of clonogenic capability and apoptosis of EC cells. In the present study, we have addressed the molecular mechanisms of anti-tumoral activity of Vorinostat. Our results demonstrate that Vorinostat treatment causes anincrease of ROS production, DNA damage and, inhibition of ROS production by addition of antioxidants dramatically reduces anti-neoplastic effects of Vorinostat.

Epigenetic deregulations is currently considered a hallmark of cancer [35]. Initially, the rationale for HDACi usage as chemotherapeutic agents was based on the observation that epigenetic deregulation was common alteration in human cancers. However, HDAC action is not restricted to the regulation of histone acetylation status, but rather regulate the acetylation status of other cytosolic or nuclear proteins that, in turn, regulate many cellular processes [36]. During the last years, several studies have evidenced that tumoricidal activity of HDACi goes beyond epigenetic regulation. Several molecular mechanisms have been proposed as mediators of anti-tumoral effects of HDACi, one of such is the generation of ROS. Our results demonstrate that EC cells show a marked increase of ROS production upon Vorinostat treatment. Although it is assumed that HDACi induces the production of ROS, the effect of addition of antioxidants on cellular outcome is still controversial. Some studies have demonstrated that inhibition of cellular antioxidant response sensitizes resistant leukemia cells lines to HDACis [37]. Accordingly, addition of antioxidants impairs HDACi cytotoxicity in acute myelogenous leukemia cells [31], [32] or colon cancer cells [38]. In contrast, other studies have demonstrated that antioxidants enhance anti-tumoral effects of HDACi on prostate cancer [39] or melanoma cells [40]. To this regard the role of antioxidants in cancer prevention and cancer therapy is still debated. From one hand, an increasing number of evidences support a cancer preventive role of antioxidants; on the other hand, antioxidants may alter or modify the efficacy of chemotherapeutic treatments [41]. Our *in vitro* and *in vivo* results demonstrate that reduction ROS levels by antioxidants results in a marked reduction of anti-tumoral activity of Vorinostat, supporting that ROS production has an important role in driving cytotoxic effects of Vorinostat in EC cells.

We have also found that ROS production correlates with increased phosphorylation of H2AX. H2AX is phosphorylated on Ser^139^ by ATM kinase in response to DNA damage-induced DNA double-strand breaks and is one of the earliest events in the DNA damage signaling and repair [42]. Vorinostat-induced DNA damage can come from two sources: it can be caused by production of ROS or, it also be independently caused by modification of DNA repair activity and chromatin remodeling factors [33]. Because we have observed that H2AX phosphorylation caused by Vorinostat is reduced by antioxidant treatment, it is likely that phosphorylation of H2AX is consequence, at least in part, of a DNA damage caused by Vorinostat-induced ROS production.

As we mentioned above, our previous results demonstrated that combinatory therapy using Vorinostat and inhibition of Caspase-8 had additive anti-tumoral effects on EC cells *in vivo*. Here, we have also studied the role of ROS on such effects. Interestingly, antioxidants failed to block reduction of tumoral growth caused by combination of Vorinostat plus caspase-8 downregulation. This result suggests that combination of such treatments is able to override the effects of antioxidants. One possibility to explain such results is that combination of caspase-8 inhibition and Vorinostatactivate alternative mechanisms of tumoral growth inhibition that are independent of ROS production. Another possible explanation is that inhibition of capase-8 impair antioxidant efficacy to block ROS activity. Nevertheless, we think that the fact of combination Vorinostat with Caspase-8 inhibition displays tumoricidal activity regardless of oxidative stress status enhances its value as possible treatment for endometrial cancer.
